# Detection of subclinical skin manifestation in patients with psoriasis and psoriatic arthritis by fluorescence optical imaging

**DOI:** 10.1186/s13075-020-02277-x

**Published:** 2020-08-18

**Authors:** A. Schmidt, A. M. Glimm, I. K. Haugen, P. Hoff, G. Schmittat, G. R. Burmester, J. Klotsche, S. Ohrndorf

**Affiliations:** 1 Department of Rheumatology and Clinical Immunology, Charité – Universitätsmedizin Berlin, Charitéplatz 1, 10117 Berlin, Germany; 2 Department of Rheumatology, Diakonhjemmet Hospital, Diakonveien 12, 0370 Oslo, Norway; 3 Deutsches Rheumaforschungszentrum (DRFZ) Berlin, A Leibnitz Institute, Berlin, Germany; 4 Institute for Social Medicine, Epidemiology and Health Economics, Charité – Universitätsmedizin Berlin, Berlin, Germany

**Keywords:** Psoriasis vulgaris, Psoriatic arthritis, Skin inflammation, Fluorescence optical imaging, Cardiovascular risk factors

## Abstract

**Objectives:**

To investigate the frequency of subclinical skin inflammation in both hands by fluorescence optical imaging (FOI) in patients with psoriasis/psoriatic arthritis (Pso/PsA) vs. rheumatoid arthritis (RA) and healthy individuals, and to correlate these findings with cardiovascular (CV) risk factors.

**Patients and methods:**

The FOI scans were analyzed retrospectively to detect clinically invisible skin enhancement (0–3 scale) in both hands without relationship to underlying joints or blood vessels. We further characterized the FOI patterns and sorted the scans into groups based on the assumed diagnosis (Pso/PsA, RA, and healthy controls), which was compared with the physician’s diagnosis. Furthermore, the associations between CV risk factors and imaging findings were investigated by regression analyses.

**Results:**

We included FOI scans of patients with Pso/PsA (*n* = 80), RA (*n* = 78), and healthy controls (*n* = 25). Subclinical skin enhancement on the back of their hands was more common in Pso/PsA (72.5%) than in RA patients (20.5%) and healthy individuals (28.0%) (*p* < 0.001). Based on the FOI pattern, the majority of patients with Pso/PsA (72.5%), RA (76.9%), and healthy controls (68.0%) were classified correctly using the physician-based diagnosis as reference (overall agreement of 74%, kappa = 0.57). No CV risk factors except body weight (kg) were associated with subclinical skin enhancement (OR 1.04, 95% CI 1.02–1.06; *p* < 0.001).

**Conclusion:**

Subclinical subdermal skin inflammation was common in Pso/PsA patients using FOI. Based on the FOI pattern, most patients with Pso/PsA and were classified with the correct diagnosis. We demonstrated an important influence of the body weight on our FOI results. FOI may be a helpful novel tool to study microcirculation in rheumatic diseases with skin involvement.

## Introduction

Psoriasis vulgaris (Pso) has a worldwide prevalence of 6–7% [1]. About 8–30% of patients with Pso develop psoriatic arthritis (PsA) [2], which are associated with increased cardiovascular morbidity and mortality [3–5]. Cardiovascular events (CV) in patients with PsA are increased up to 43% compared to the general population [5]. The increase in mortality seems to be associated with severity of PsA [6] and disease duration [7, 8]. Furthermore, PsA patients reveal an increased prevalence of CV risk factors such as hypertension [9, 10], diabetes mellitus [6, 10], hyperlipidemia [10, 11], and the full metabolic syndrome [12–16]. Accordingly, a significantly increased prevalence of metabolic syndrome in patients with PsA compared to rheumatoid arthritis (RA) patients was demonstrated recently [12], although comparable inflammatory pathways can be seen in both diseases [17].

Approaches to explain these phenomena have already been explored in various studies with a different focus. Of particular interest are studies that link inflammatory pathways between skin inflammation and CV disease-triggering inflammatory modulators such as T cells, T-helper cells [18–22], tumor-necrosis-factor alpha (TNF-alpha), and interleukin (IL)-6 or IL-17 [23, 24]. Karbach et al. showed an association between dermal IL-17A and systemic vascular dysfunction [23]. Wang et al. also presented that psoriatic skin inflammation alone could predict aortic root inflammation after 1 year of observation [22]. In addition to these in vitro studies and those using mouse models, it has been shown that patients with PsA have a significantly elevated arterial stiffness compared to healthy controls and that arterial stiffness correlates with the duration of joint involvement [25]. Elevated IL-17 levels in the serum of Pso-induced mice have already been measured and a link between elevated serum levels and the severity of Pso was presented [26, 27]. Thus, inflamed vessels and elevated levels of mediators connecting them with skin inflammation were represented, but to our knowledge, there has never been an attempt to visualize the existing subclinical skin inflammation. Fluorescence optical imaging (FOI) was recently introduced as an additional imaging technique in the diagnostics of systemic inflammatory joint diseases, visualizing disturbed microcirculation in both hands. Inflammation causes increased vasodilation and capillary permeability in the affected areas. As a result, the injected fluorescence dye will accumulate in inflamed areas and leads to a signal enhancement in the involved joints. Werner et al. and Fischer et al. showed that FOI correlates well with clinical examination, MRI, or ultrasound findings in patients with arthritis [28, 29]. Furthermore, it has been shown that FOI is able to visualize changes in joint inflammation during anti-rheumatic therapy [30, 31] and that it can even detect subclinical synovitis in patients with inflammatory arthritis [32]. It was not only used in diagnostics and monitoring of RA and other arthritic diseases, but also for the detection of a disturbed microcirculation and prediction of digital ulcers in systemic sclerosis (SSc) [33, 34]. Pfeil et al. used FOI in SSc patients to detect inflammation of the soft tissue and monitor the effectiveness of treatment [35]. To our knowledge, no previous study has used FOI to visualize psoriatic skin inflammation accompanying PsA.

Therefore, our aim was to explore the frequency and patterns of subclinical skin inflammation in both hands in patients with Pso/PsA by FOI in comparison with RA and healthy individuals and to correlate these findings with CV risk factors or events.

### Patients and methods

#### Included patient cohorts

Eighty patients diagnosed with Pso/PsA, 80 patients diagnosed with RA, and 28 healthy controls were selected from different cohort studies. We included patients with a definite diagnosis of Pso (diagnosed by a dermatologist), PsA (diagnosed by a dermatologist and a rheumatologist), or RA (diagnosed by a rheumatologist) and at least one FOI examination.

Exclusion criteria were observed psoriatic lesions or other obvious non-psoriatic wounds on the hands, which were documented for each patient via illustration. Patients who were missing these illustrations were excluded. Furthermore, a glomerular filtration rate (GFR) below 60 ml/min, hyperthyroidism, breastfeeding, pregnancy, and age below 18 years were general exclusion criteria to perform the FOI examination (see Additional file 1 for the flow chart concerning the patient recruitment).

Ethical committees in Germany gave the required ethical approvals for the respective studies (128/13 EK, 127/13 EK, EA1/025/1, EA1/193/10, EA1/269/13). All patients from the different studies were originally recruited in the department of rheumatology (i.e., outpatient clinic and day unit) of the Charité – Universitätsmedizin Berlin. All included patients got written and oral information about the study and signed the informed consent.

#### FOI method and examination

Ten seconds after start of the examination, the fluorescent dye Indocyanine green (ICG) is intravenously administered (0.1 mg/kg of the body weight). After stimulation of the dye by light of the near-infrared spectrum, a special charge-coupled-device (CCD) camera detects occurring light emission and presents it visually as enhancements in both hands. During the 6 min of examination, one image per second is recorded adding up to a cluster of 360 pictures in total. To assess the examination, there are different methods described. The most common method to analyze joint inflammation on FOI scans is the *Berlin method*, which classifies the image sequence into three phases. Depending on the signal in the fingertips, the signals in each phase are evaluated regarding intensity, size, and shape and are classified into three grades (0–3) according to the semi-quantitative FOI Activity Score (FOIAS) [29, 36–38]. Using this assessment, joints showing hypervascularization can be detected and, thus, the activity of inflammation can be estimated.

#### Detecting subclinical skin enhancement

In order to detect potential subclinical skin enhancement, we developed a new semi-quantitative (0–3) score to describe the degree of the skin enhancement. The score concentrates on the first part of the image sequence (0–120 s) since we noticed that the enhancements were only visible in this time frame.

The FOI scans were randomized and blinded and then evaluated according to our defined criteria using example images in an atlas as reference (Supplementary File). The images were scored by one reader (AS). In order to analyze the interreader reliability, a second reader (SO) scored a sample of 90 randomized and blinded cases.

These defined criteria had to be fulfilled before evaluation:

- At least 90% of the respective hand must be green flooded in the respective time frame
- The enhancement suspicious of subclinical skin enhancement was assumed to be localized on the back of the hand without any relationship to a joint, or blood vessel.
- The enhancement had to be at least yellow with red spots in intensity to be considered as such.

**Fig. 1:**
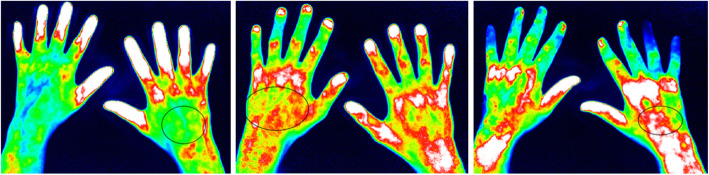
FOIAS modified for subclinical skin enhancement. Left = grade 1, middle = grade 2, right = grade 3. The marked areas on the back of the hand show areas with subclinical enhancement detected by FOI. Left picture: The enhancement is mostly yellow on green ground classified as grade 1. Middle picture: The enhancement is red with minimal white signals in it classified as grade 2. Right picture: The enhancement in the marked area shows more white than red signals which represents grade 3

The color intensity was evaluated semi-quantitatively (grade 0–3) similar to the FOIAS (Fig. 1):

- ◦ *0* = no signal enhancement
- ◦ *1* = enhancement varies from yellow to red and can reach red with yellow spots, red covers ≤ 50% of the enhanced/affected joint area
- ◦ *2* = the signal intensity shows strong red color intensity and can also include white signals, and white covers ≤ 50% of the enhanced/affected joint area
- ◦ *3* = the signal intensity shows white color intensity, and white covers > 50% of the enhanced/affected joint area

To ensure that the detected skin enhancement did not show a flooding vessel, it was recommended to read the same sequence with the XiraView 3.7 “Temperature” filter. This is a particularly effective way of displaying vessels and it is possible to check the area in question for them. (Examples in Supplementary Figure S2).

In order to describe where the lesions were most frequently found, the back of the hand was divided into five regions (Figure in Additional file 2 FOI Atlas):

- ◦ *Region 1*: below the 2nd metacarpal joint (MCP2) and next to MCP1, not touching any of them or the thumb base joint
- ◦ *Region 2*: below MCP3 down to the middle of the back of the hand
- ◦ *Region 3*: below MCP4 down to the middle of the back of the hand
- ◦ *Region 4*: below MCP5 down to the middle of the back of the hand
- ◦ *Region 5*: above the wrist joints up to the middle of the back of the hand

**Fig. 2:**
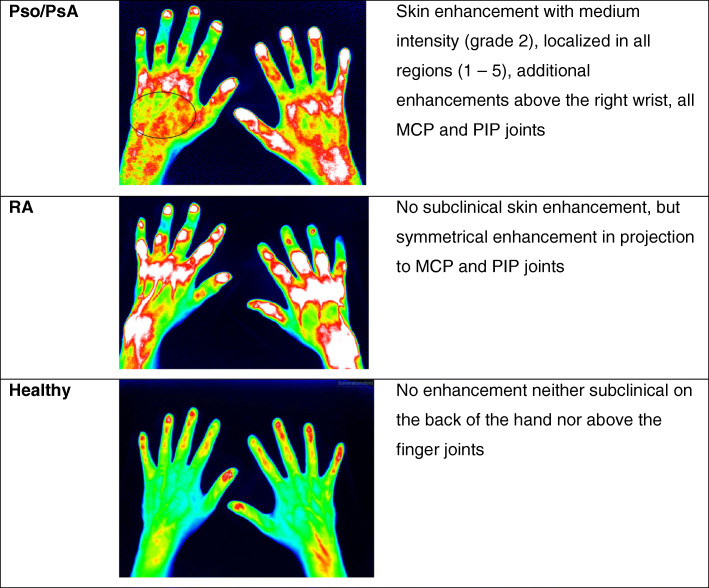
Examples for categories Pso/PsA, RA, and healthy controls

An FOI-based diagnosis was made based on the subclinical skin enhancement on the dorsum of the hand and enhancement of joints and tendons (Fig. 2):

- ◦ *Pso/PsA*: subclinical skin enhancement and (in case of PsA) further enhancements above the joints (according to FOIAS)
- ◦ *RA*: No subclinical skin enhancement (but enhancement above the joints according to FOIAS)
- ◦ *Healthy control*: No subclinical skin enhancement (and no further enhancements above the joints according to FOIAS)

We further characterized the FOI patterns and sorted the scans into groups based on the assumed diagnosis (Pso/PsA, RA, and healthy controls), which was compared with the physician’s diagnosis. The physician’s diagnosis was extracted as the final diagnosis in the last available physician’s letter.

To identify possible influences on our FOI findings, we tested potential influencing factors (e.g., CV risk factors, sex, disease duration) for both false positive and false negative results. False positive results refer to detected skin enhancement in patients who, according to our definition, should not show any skin enhancement (i.e., RA patients and healthy controls). Accordingly, false negative results are non-existing skin enhancements in Pso/PsA, since we hypothesized that all Pso/PsA patients should show subdermal skin enhancements.

Furthermore, we investigated potential association between CV factors and imaging findings. CV risk factors such as hypertension, metabolic syndrome, or smoking status were recorded and correlated to the imaging results.

### Statistical analysis

Sociodemographic and clinical characteristics were compared by analysis of variance for continuously distributed variables or a chi-square test for categorical variables between the three groups Pso/PsA, RA, and healthy controls. The concordance between the rate of FOI positive cases and the FOI diagnosis by one reader with the clinical diagnosis was determined. Based on the gold standard of the clinical diagnosis, the rates of true positive, true negative, false positive, and false negative FOI results were calculated. Multinomial logistic regression analyses were conducted to model the association between the four groups true positive, true negative, false positive, and false negative with clinical and sociodemographic characteristic as predictor variables. The reference group was set to positive classified patients in the multinomial logistic regression model. In addition, the association of clinical and sociodemographic variables with the outcome FOI enhancement was estimated by means of binary logistic regression model. The interreader agreement was investigated in 90 randomly selected and blinded patients between the ratings of the two readers AS and SO. Absolute agreement rates and the kappa coefficient were calculated to evaluate agreement. A *p* value of < 0.05 was considered statistically significant. Statistical analyses were conducted with the statistical software STATA 12.1 (StataCorp. 2011. Stata Statistical Software: Release 12. College Station, TX: StataCorp LP).

## Results

### Demographic and clinical characteristics

Demographic and clinical details of patient cohorts are shown in Table 1.

**Table 1:** Sociodemographic and clinical characteristics

	Pso/PsA		RA		Healthy	
	*n* = 80		*n* = 78		*n* = 25	
**Sex,** ***n*** **(%)**						
Female	56	70.0	59	75.6	16	64.0
**Age in years, mean (SD)**	48.5 (12.32)		52.7 (13.23)		29.0 (10.13)	
**BMI in kg/m**^**2**^**, mean (SD)**	27.6 (5.9)		26.1 (4.7)			
**Disease duration in years, mean (SD)**	8.5 (10.8)		2.4 (4.7)			
**Anti-CCP, mean (SD)**	12.6 (13.4)		298.8 (401.7)			
Anti-CCP ≥ 20 U/ml, *n* (%)	9	11.8	48	63.2		
**RF (IgA) in U/ml, mean (SD)**	6.9 (7.2)		98.1 (163.0)			
IGA ≥ 20 U/ml, *n* (%)	3	3.9	34	44.7		
**RF (IgM) in U/ml, mean (SD)**	4.2 (4.5)		87.0 (146.1)			
IGM ≥ 20 U/ml, *n* (%)	3	3.9	34	44.7		
**CRP in mg/l, mean (SD)**	3.9 (5.5)		13.2 (16.9)			
**ESR in mm/h, mean (SD)**	17.2 (11.1)		34.8 (23.3)			
HLA-B27 positive, *n* (%)	2	15.4	0	0.0		
DAS28, mean (SD) (range 0–10)	3.1 (1.4)		5.1 (1.3)			
TJC-28, mean (SD) (range 0–28)	5.1 (4.5)		8.7 (6.9)			
SJC-28, mean (SD) (range 0–28)	1.7 (2.4)		5.1 (4.3)			
Physicians’ global (VAS), mean (SD) (range 0–100)	2.9 (2.0)		4.7 (1.6)			
PASI, mean (SD)	2.9 (3.2)					
Body surface area, mean (SD)	8.5 (9.6)					
Patient’s global (VAS), mean (SD) (range 0–100)	5.0 (2.2)		5.7 (2.1)			

The female predominance was larger in the patient groups than among the healthy controls. Similarly, the RA and Pso/PsA patients were older than the healthy controls.

The CRP levels were higher in the RA group than in the Pso/PsA group. Similarly, the RA patients had higher disease activity scores than the Pso/PsA subgroup. Concerning the medication, more RA than Pso/PsA patients received conventional synthetic disease-modifying anti-rheumatic drugs (csDMARD) and glucocorticoids, whereas more Pso/PsA than RA patients received biological DMARD (bDMARD) (Supplementary Table S1). These data were not obtained in the healthy subgroup.

Regarding the history of cardiovascular risk factors or diseases, there were no significant differences between patients with Pso/PsA and RA (Supplementary Table S2).

### Association between FOI results and diagnosis

The frequency of FOI-defined skin enhancement was considerably higher in patients with Pso/PsA than in patients with RA or healthy controls (Table 2).

**Table 2:** Agreement between detected skin enhancements and physician’s diagnosis

	Pso/PsA		RA		Healthy		
	*n* = 80		*n* = 78		*n* = 25		
	*N*	%	*N*	%	*N*	%	***p*** value
**FOI positive**	58	72.5	16	20.5	7	28.0	**< 0.001**
**FOI Diagnosis**							
Pso / PsA	58	72.5	16	20.5	7	28.0	
RA	16	20.0	60	76.9	1	4.0	
Healthy	6	7.5	2	2.6	17	68.0	
**If FOI positive: FOIAS grades**							0.255
1	13	22.4	4	25.0	0	0.0	
2	42	72.4	9	56.3	6	85.7	
3	3	5.2	3	18.8	1	14.3	
**If FOI positive (*n*** **= 81): localization**							
Region 1	26	32.5	12	15.4	4	16.0	**0.026**
Region 2	28	35.0	5	6.4	6	24.0	**< 0.001**
Region 3	8	10.0	7	9.0	4	16.0	0.598
Region 4	23	28.8	12	15.4	5	20.0	0.123
Region 5	39	48.8	10	12.8	4	16.0	**< 0.001**

The FOI-based diagnoses were compared with the physician’s diagnoses. Fifty-eight of the 80 Pso/PsA patients (72.5%) were correctly classified as Pso/PsA. Of 78 RA patients, 60 (76.9%) were classified as RA, and of 25 healthy controls, seventeen (68.0%) were classified as healthy resulting in an overall agreement of 74% (kappa = 0.57). Subdermal skin enhancement was mostly detected in region 1 and in region 5 (Table 2).

### Influences on false negative/false positive results

Correlates of false negative and false positive findings were analyzed in patients with Pso/PsA and RA only; healthy controls were excluded from this analysis due to non-available parameters. Body weight was significantly associated with the correct classification of patients by FOI with respect to the physician’s diagnosis. Patients with a true negative FOI finding (i.e., no skin enhancement in RA patients) had a significantly lower weight (OR 0.96, CI 0.94; 0.99, *p* = 0.003) as compared to true positive classified patients. Likewise, the likelihood of receiving a false negative FOI finding (i.e., no skin accumulations in Pso/PsA patients) was also associated with a lower body weight (OR 0.95, CI 0.92; 0.99, *p* = 0.010). False positive FOI findings were more likely reported for patients with metabolic syndrome (*n* = 1, 1.8% among true positive findings versus *n* = 3, 18.8%, OR 12.7; CI 1.2; 131, *p* = 0.033 false positive findings). Patient global assessment was significantly higher for patients who were classified as false positive (mean 6.7, SD 1.8, OR 1.45; CI 1.09; 1.94; *p* = 0.012 versus mean 5.1, SD 2.2) compared to truly positive classified patients.

In addition, patients with false positive results tended to show a short disease duration (mean 1.9 years). With each more year of disease duration, the probability to show a false positive signal decreased by 14% (*p* = 0.049, OR 0.86, CI 0.75; 1.00). Patients with false negative results tended to show a long disease duration (mean 9.3 years). However, the difference was not significant (*p* = 0.149).

### Association to CV risk factors

To test our secondary hypothesis in patients with Pso/PsA and RA, we additionally investigated potential associations of FOI findings with CV risk factors. As mentioned before, the body weight appeared to have an influence on the FOI results. Furthermore, a higher Patients Global Rating correlated with a higher probability of showing skin enhancement. In the overall cohort, this correlation was not significant (*p* = 0.665, OR 1.03, CI 0.89; 1.02). In the subgroup of RA patients, however, this association was significant (OR 1.33, CI 1.00; 1.77, *p* = 0.047).

Except BMI, further associations with FOI findings were not found for other CV risk factors or pre-existing cardiovascular diseases (data not shown).

### Interreader agreement

The interreader reliability for the overall sample concerning the diagnosis was fair (κ = 0.35, absolute agreement 59.6%). However, a significantly higher interreader agreement was found in the detection of Pso/PsA and RA patients. Thus, both readers had an agreement of 63.4% in the classification of Pso/PsA and an agreement of 65.4% in the classification of RA. The correspondence in the classification of healthy controls was significantly lower, only 41.2% agreement.

The agreement of reader 1 with the actual diagnosis in the physician’s report for the overall cohort was moderate with κ = 0.57 (absolute agreement 73.8%).

## Discussion

FOI has been used for various questions and diseases in rheumatology. For example, investigation was performed in the detection of inflamed joints in RA [28, 30, 39–41]. Following studies concentrated on the examination of OA with different inflammatory patterns [36] or decreased blood flow in systemic sclerosis [33, 34]. To our knowledge, we are the first group to investigate potential subclinical inflammation of the skin in Pso/PsA patients. Since skin inflammation, increased rate of vessel inflammation [42], and arterial stiffness [25] has been shown in patients with Pso/PsA, we hypothesized that ICG-based FOI would be a valuable method to visualize impaired microcirculation in Pso/PsA [37].

In our analysis, 72.5% of Pso/PsA patients showed subclinical skin enhancements, whereas only 20.5% of RA patients and 28.0% of healthy controls exhibited comparable enhancement patterns. Thus, significantly more patients diagnosed with PsA or Pso presented subdermal skin enhancements in FOI. This confirmed our primary hypothesis and shows that it is possible to visualize subclinical skin inflammation by FOI.

However, there were some false positive (i.e., detected skin enhancements in RA patients and healthy controls) and false negative (i.e., no enhancements in Pso/PsA patients) findings. We hypothesized that the disease duration could be one influencing factor to false positive results. There was a difference between the disease duration in patients with true positive (skin enhancements in Pso/PsA patients) and false positive findings. There was a shorter disease duration in those patients with false positive results. A higher vessel permeability due to vessel inflammation could therefore be present in acute, newly appeared inflammatory arthritic disease such as RA.

Another influencing factor was the patient’s body weight. With increasing body weight, more enhancement was found leading to higher chance of correct classification of Pso/PsA patients, but also higher chance of misclassification of RA and healthy controls. Overweight and obesity are associated with a systemic low–grade inflammation, which might have influenced our results.

Our secondary hypothesis was that we are able to correlate the FOI results with cardiovascular risk factors since these risk factors are well known to be specifically high in Pso/PsA patients [5, 9–16]. However, in our study, only the body weight had an influence on the FOI results. In this respect, we did not measure the blood pressure directly before and after the FOI scan and did not do a full metabolic status on the patients. Moreover, we did not do a follow-up, in which possible further cardiovascular risks or events, that occurred in the meantime, might have turned up. Also, this was a retrospective study and the results need to be investigated in a prospective, larger setting.

Nevertheless, we could show that FOI might be an interesting tool to study skin involvement in rheumatic diseases. Moreover, if further validated, it might be a complementary diagnostic tool to recognize an early undifferentiated arthritis as a beginning psoriasis arthritis. But it could not only be interesting in diagnostics, but also in therapy monitoring. Due to the fact that therapy monitoring according to the Treat-to-Target (T2T) strategy becomes more of interest in Pso/PsA therapy, there is an increasing need for objective monitoring tools, especially since targeted remission assessed by the DAPSA can still show a residual cutaneous inflammation [43, 44]. Our new method of visualizing subdermal skin inflammation may in future become a valuable addition to the DAPSA in monitoring “real” psoriatic skin remission.

### Limitations

We are aware of some limitations concerning our study design. Due to the retrospective design of the study, some of the data were incomplete. Data from healthy controls were collected only to a limited extent. In addition, because of the limited number of patients that received an FOI scan, we included all patients that met our inclusion and exclusion criteria. Therefore, our subgroups could not be matched by age, sex, or disease duration, which makes direct comparability of the subgroups difficult.

Additionally, the interreader reliability was fair. Like all imaging techniques, FOI is dependent on the experience of the reader. Since this study represents a first step into the detection of potential skin enhancement, there was no experience when we started to develop this method. Reader 1 (AS) who scored all FOI scans achieved a higher agreement between FOI results and physician’s diagnosis than Reader 2 (SO) achieved between her FOI results and the ones of Reader 1. We are confident that the interrater reliability would increase with increasing experience and better-defined influencing factors.

## Conclusion

In conclusion, it was possible to detect potential subdermal skin inflammation in Pso/PsA patients by FOI. Using FOI, we were able to categorize the majority of Pso/PsA and RA patients correctly according to the physician-based diagnosis. Although we could not describe a correlation between subdermal skin enhancement and cardiovascular risk factors, we found an influence of the body weight, which should be further investigated. Thus, FOI may be a helpful novel tool to study microcirculation in rheumatic diseases with skin involvement.

## Supplementary information

**Additional file 1:** **Figure S1.** Flow-Chart: Patient recruitment. **Figure S2.** Subdermal skin enhancement read in “Temperature mode”. **Table S1.** Current medication. **Table S2.** Cardiovascular risk factors.

**Additional file 2:** FOI Atlas_subclinical skin enhancement.

## Data Availability

The datasets used and/or analyzed during the current study are available from the corresponding author on reasonable request.

## Abbreviations

Anti-CCP: Anti-cyclic citrullinated peptide
bDMARD: Biological disease-modifying anti-rheumatic drugs
BMI: Body mass index
CCD: Charge-coupled-device
CHD: Coronary heart disease
CI: Confidence interval
COPD: Chronic obstructive pulmonary disease
CRP: C-reactive protein
csDMARD: Conventional synthetic disease-modifying anti-rheumatic drugs
CV: Cardiovascular events
CVD: Cardiovascular disease
DAS28: Disease activity score
e.g.: Exempli gratia, Latin for “for example”
ESR: Erythrocyte sedimentation rate
FDG-PET/CT: [18F]-fluorodeoxyglucose positron emission tomography-computed tomography
FOI: Fluorescence optical imaging
FOIAS: FOI activity score
GFR: Glomerular filtration rate
HDL / LDL: High-density lipoprotein / low-density lipoprotein
i.e.: “id est”
ICG: Indocyanine green
IL-17A: Interleukin-17A
MCP: Medio-carpo-pharyngeal (joint)
OR: Odds ratio
PASI: Psoriasis Area and Severity Index
PsA: Psoriatic arthritis
Pso: Psoriasis vulgaris
RA: Rheumatoid arthritis
RF: Rheumatoid factor
SD: Standard deviation
SJC: Swollen joint count
T2T: Treat-to-target
TIA: Transient ischemic attack
TJC: Tender joint count
TNF-α: Tumor-necrosis-factor alpha
