# Supplementary material for: Detection of subclinical skin manifestation in patients with psoriasis and psoriatic arthritis by fluorescence optical imaging
Source: Arthritis Res Ther. 2020 Aug 18;22:192. doi: 10.1186/s13075-020-02277-x (PMC7433190; doi:10.1186/s13075-020-02277-x)
Supplement: Supplementary file 1 — Additional file 1: Figure S1. Flow-Chart: Patient recruitment. Figure S2. Subdermal skin enhancement read in “Temperature mode”. Table S1. Current medication. Table S2. Cardiovascular risk factors. [file 13075_2020_2277_MOESM1_ESM.docx]

# Supplementary figures and tables

***Figure S1: Flow-Chart: Patient recruitment***

PsA Patients

N = 95

Arthromark

Healthy Controls

N = 28

RA patients

N = 80

Pso Patients

N = 60

noted lesions on the hands at the time of the FOI scan or lesions on the hands that were not safely excluded

N = 75

All patients assessed for inclusion and exclusion criteria

FOI scan defect, therefore not analyzable

N = 5 (RA=2, healthy=3)

Final cohort

N = 183

***Figure S2: Subdermal skin enhancement read in “Temperature mode”***

| 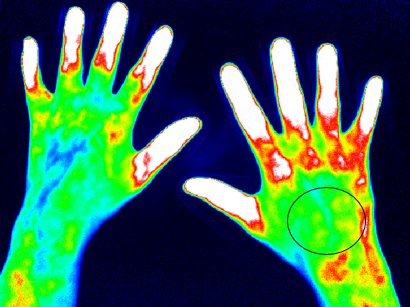 | 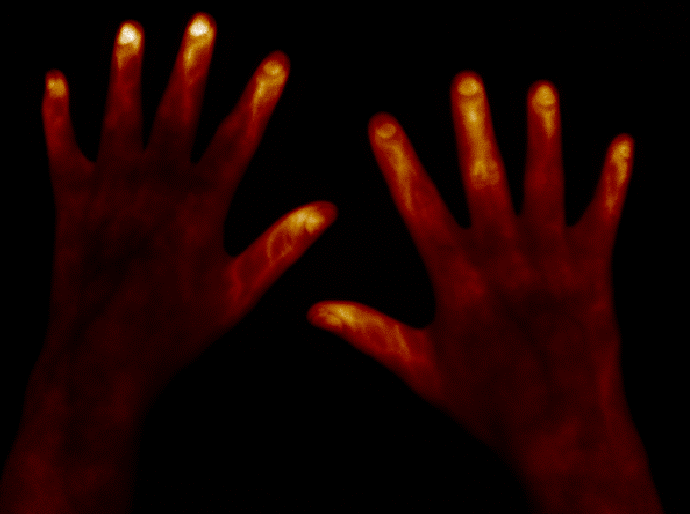 |
| --- | --- |
| 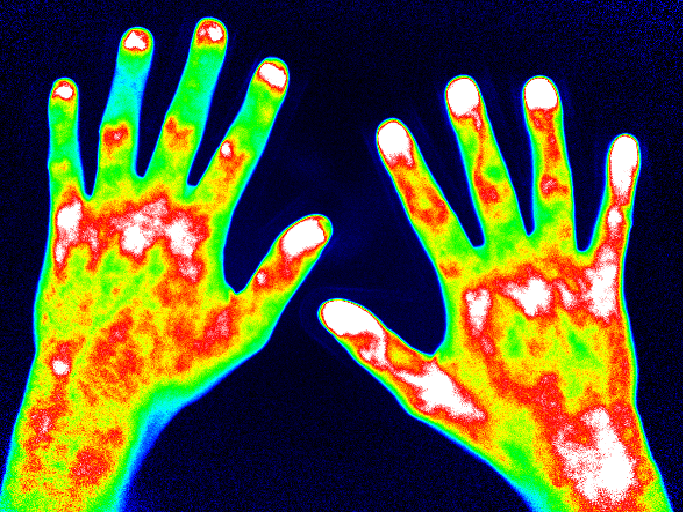 | 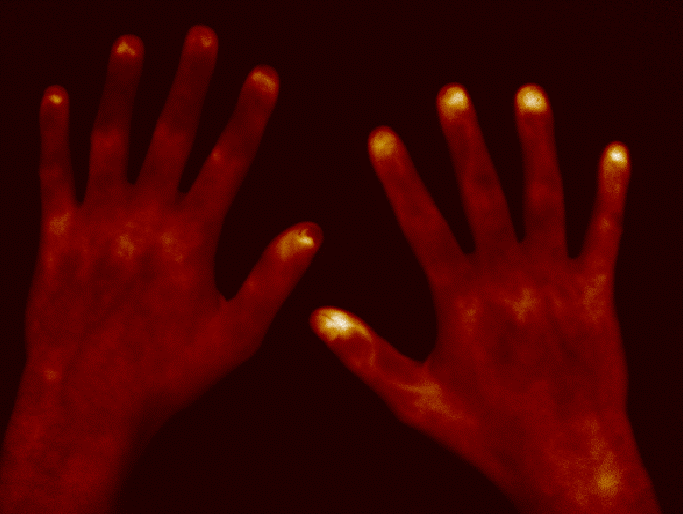 |
| 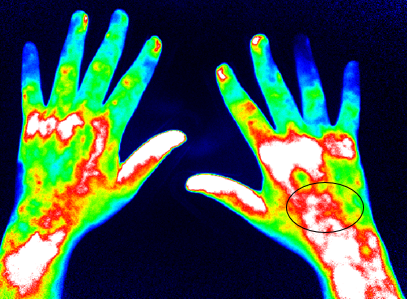 | 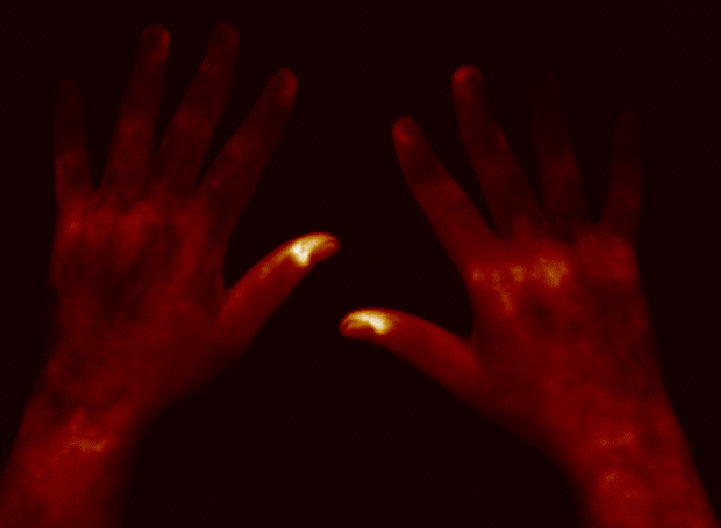 |

*Comparison of the two possible modes to control suspected areas of subdermal skin enhancement for underlying blood vessels. The marked areas show no signs of blood vessels in the “Temperature mode”, which confirms that the enhancement is probably located in the (sub)-dermis.*

***Table S1:*** *Current medication*

|  | | **Pso / PsA** | | **RA** | | | **Healthy** | |
| --- | --- | --- | --- | --- | --- | --- | --- | --- |
|  | | n=80 | | n=78 | | | n=25 | |
|  | | N | % | N | N | | % | N |
| **Current medication** | |  |  |  |  | |  |  |
|  | none | 37 | 46.3 | 9 | 11.5 | |  |  |
|  | **csDMARD** | **39** | **48.8** | **61** | **78.2** | |  |  |
|  | **bDMARD** | **35** | **43.8** | **14** | **18.0** | |  |  |
|  | NSAID | 38 | 47.5 | 44 | 56.4 | |  |  |
|  | Opioid | 8 | 10.0 | 4 | 5.1 | |  |  |
|  | PPI | 25 | 31.3 | 47 | 60.3 | |  |  |
|  | Antihypertensiva | 23 | 28.8 | 51 | 65.4 | |  |  |
|  | Antidiabetic therapy | 7 | 8.8 | 5 | 6.4 | |  |  |
|  | Insuline | 6 | 7.5 | 4 | 5.1 | |  |  |
|  | Platelet aggregation inhibitors | 2 | 2.5 | 8 | 10.3 | |  |  |
|  | Calcium antagonists | 9 | 11.3 | 6 | 7.7 | |  |  |
|  | Lipid lowering therapy | 4 | 5.0 | 9 | 11.5 | |  |  |
|  | Thyreostatics | 1 | 1.3 | 0 | 0.0 | |  |  |
|  | L-thyroxine | 7 | 8.8 | 8 | 10.3 | |  |  |
|  | Antidepressive treatment | 4 | 5.0 | 0 | 0.0 | |  |  |
|  | Supplements | 12 | 15.0 | 37 | 47.4 | |  |  |
|  | **Glucocorticoids** | **5** | **6.3** | **42** | **53.9** | |  |  |
|  | Inhaled glucocorticoids | 1 | 1.3 | 1 | 1.3 | |  |  |
|  | Inhalative Betamimetika | 3 | 3.8 | 1 | 1.3 | |  |  |
|  | Urikostatika | 1 | 1.3 | 1 | 1.3 | |  |  |
|  | Topical psoriasis therapy | 17 | 21.3 | 0 | 0.0 | |  |  |
|  | Diuretics | 3 | 3.8 | 5 | 6.4 | |  |  |
|  | Antihistamines | 1 | 1.3 | 0 | | 0.0 |  | |
|  | Anticonvulsants | 3 | 3.8 | 1 | | 1.3 |  | |
|  | Antiemetics | 1 | 1.3 | 0 | | 0.0 |  | |
|  | Triptans | 1 | 1.3 | 0 | | 0.0 |  | |
|  | Contraceptives | 1 | 1.3 | 1 | 1.3 | |  |  |
|  | Parathyroid hormone analogs | 1 | 1.3 | 0 | 0.0 | |  |  |
|  | Myotonolytics | 0 | 0.0 | 1 | 1.3 | |  |  |
|  | Alpha blockers | 0 | 0.0 | 3 | 3.9 | |  |  |
|  | Aldosterone antagonists | 0 | 0.0 | 1 | 1.3 | |  |  |
|  | Progesterone | 0 | 0.0 | 1 | 1.3 | |  |  |

***Table S2:*** *Cardiovascular risk factors*

|  | | **Pso / PsA** | | **RA** | | **Healthy** | |
| --- | --- | --- | --- | --- | --- | --- | --- |
|  | | n=80 | | n=78 | | n=25 | |
|  | | N | % | N | N | % | N |
| **Cardiovascular Risk factors** | |  |  |  |  |  |  |
|  | No comorbid disease | 35 | 44.9 | 30 | 38.5 |  |  |
|  | Hypertension | 29 | 37.2 | 43 | 55.1 |  |  |
|  | Metabolic syndrome | 1 | 1.3 | 10 | 12.8 |  |  |
|  | Adipositas | 6 | 7.7 | 3 | 3.9 |  |  |
|  | Prior thyroidectomy/ hypothyroidism | 9 | 11.5 | 5 | 6.4 |  |  |
|  | Angina pectoris | 11 | 14.1 | 8 | 10.3 |  |  |
|  | Venous insufficiency/ varicosis | 2 | 2.6 | 3 | 3.9 |  |  |
|  | Hyperlipidemia | 5 | 6.4 | 17 | 21.8 |  |  |
|  | Coronary artery disease | 0 | 0.0 | 3 | 3.9 |  |  |
|  | Prior myocardial infarction | 0 | 0.0 | 3 | 3.9 |  |  |
|  | Heart failure | 0 | 0.0 | 2 | 2.6 |  |  |
|  | Prior stroke/ TIA | 0 | 0.0 | 2 | 2.6 |  |  |
|  | Obstructive sleep apnea syndrome | 0 | 0.0 | 1 | 1.3 |  |  |
|  | Graves' disease | 1 | 1.3 | 0 | 0.0 |  |  |
|  | Diabetes mellitus | 10 | 12.8 | 8 | 10.3 |  |  |
|  | Prior deep vein thrombosis | 3 | 3.9 | 0 | 0.0 |  |  |
|  | Atrial fibrillation | 1 | 1.3 | 0 | 0.0 |  |  |
|  | Cancer | 1 | 1.3 | 1 | 1.3 |  |  |
|  | COPD | 0 | 0.0 | 1 | 1.3 |  |  |
|  |  |  |  |  |  |  |  |
|  | HDL in mg/dl. mean (SD) | 60.7 (17.12) | | 58.1 (21.29) | |  | |
|  | LDL in mg/dl. mean (SD) | 135.9 (73.24) | | 131.9 (50.67) | |  | |
|  | Total cholesterol in mg/dl. mean (SD) | 213.7 (87.80) | | 213.6 (48.68) | |  | |
|  | Triglyceride in mg/dl. mean (SD) | 130.6 (98.94) | | 161.2 (96.01) | |  | |
|  |  |  |  |  |  |  |  |
