# Supplementary material for: Detection of subclinical skin manifestation in patients with psoriasis and psoriatic arthritis by fluorescence optical imaging
Source: Arthritis Res Ther. 2020 Aug 18;22:192. doi: 10.1186/s13075-020-02277-x (PMC7433190; doi:10.1186/s13075-020-02277-x)
Supplement: Supplementary file 2 — Additional file 2: FOI Atlas_subclinical skin enhancement. [file 13075_2020_2277_MOESM2_ESM.pptx]

## Slide 1
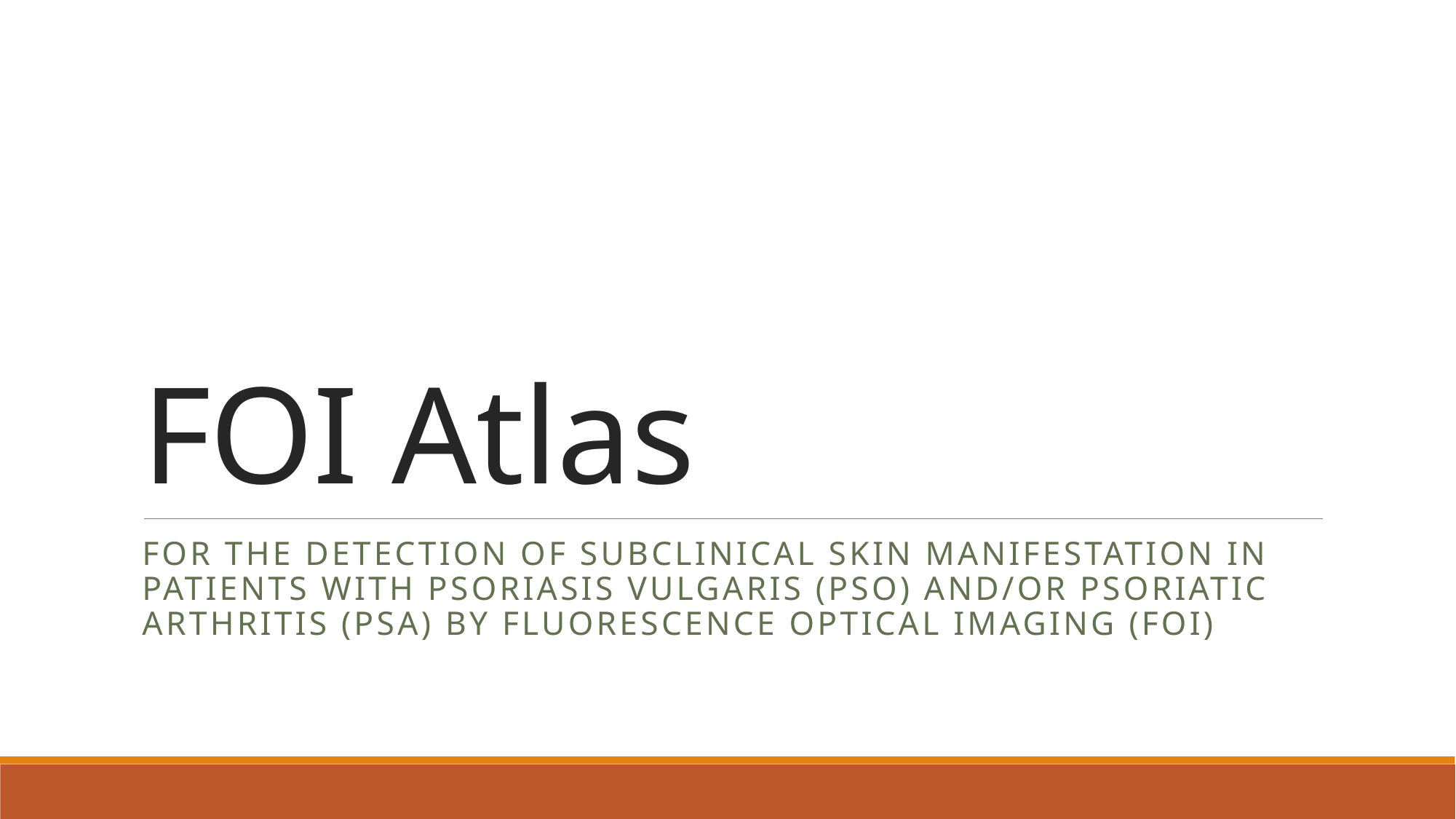

# FOI Atlas
For the detection of subclinical skin manifestation in patients with psoriasis vulgaris (Pso) and/or Psoriatic arthritis (PsA) by fluorescence optical imaging (FOI)

## Slide 2
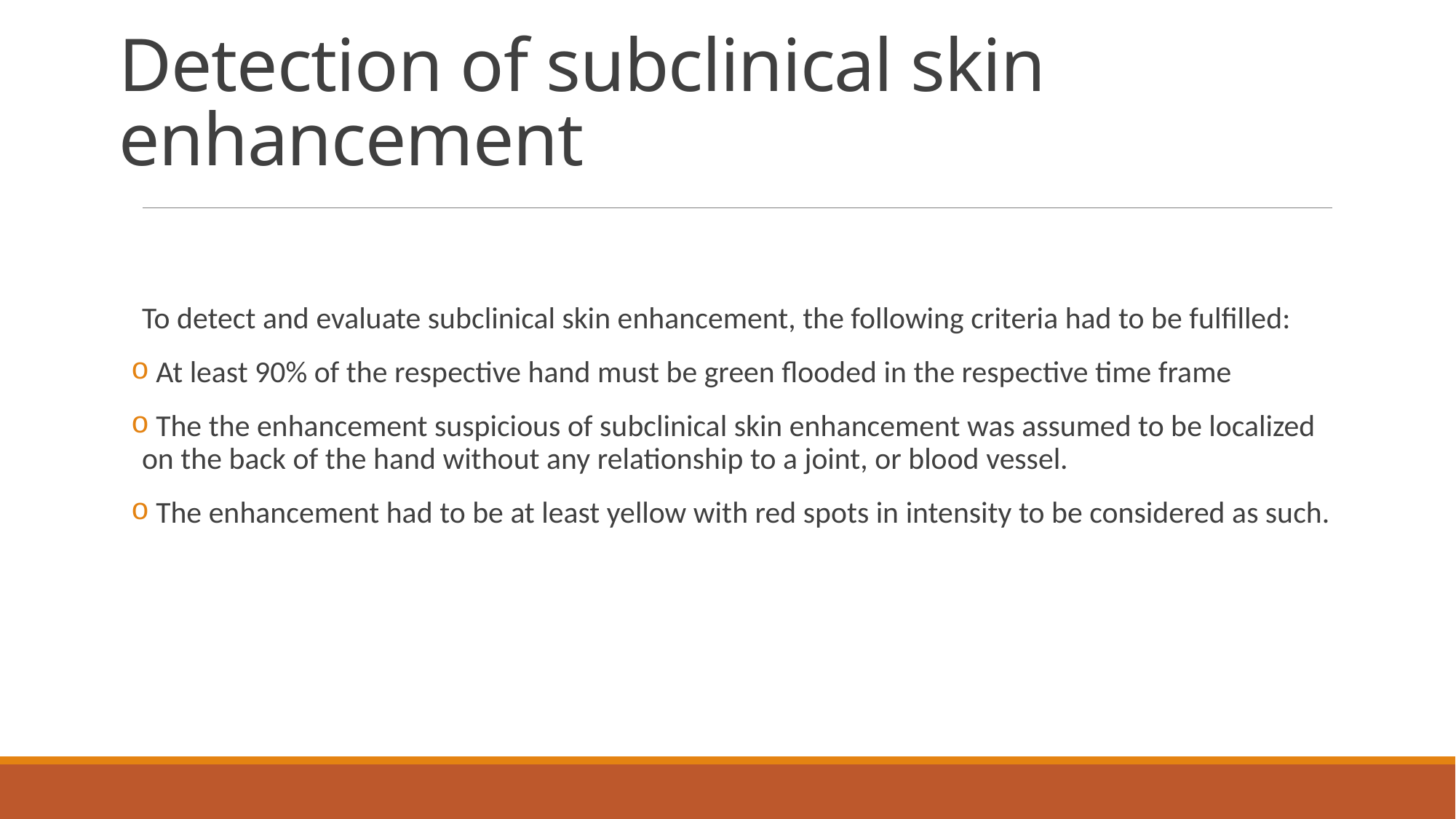

# Detection of subclinical skin enhancement
To detect and evaluate subclinical skin enhancement, the following criteria had to be fulfilled:
 At least 90% of the respective hand must be green flooded in the respective time frame
 The the enhancement suspicious of subclinical skin enhancement was assumed to be localized on the back of the hand without any relationship to a joint, or blood vessel.
 The enhancement had to be at least yellow with red spots in intensity to be considered as such.

## Slide 3
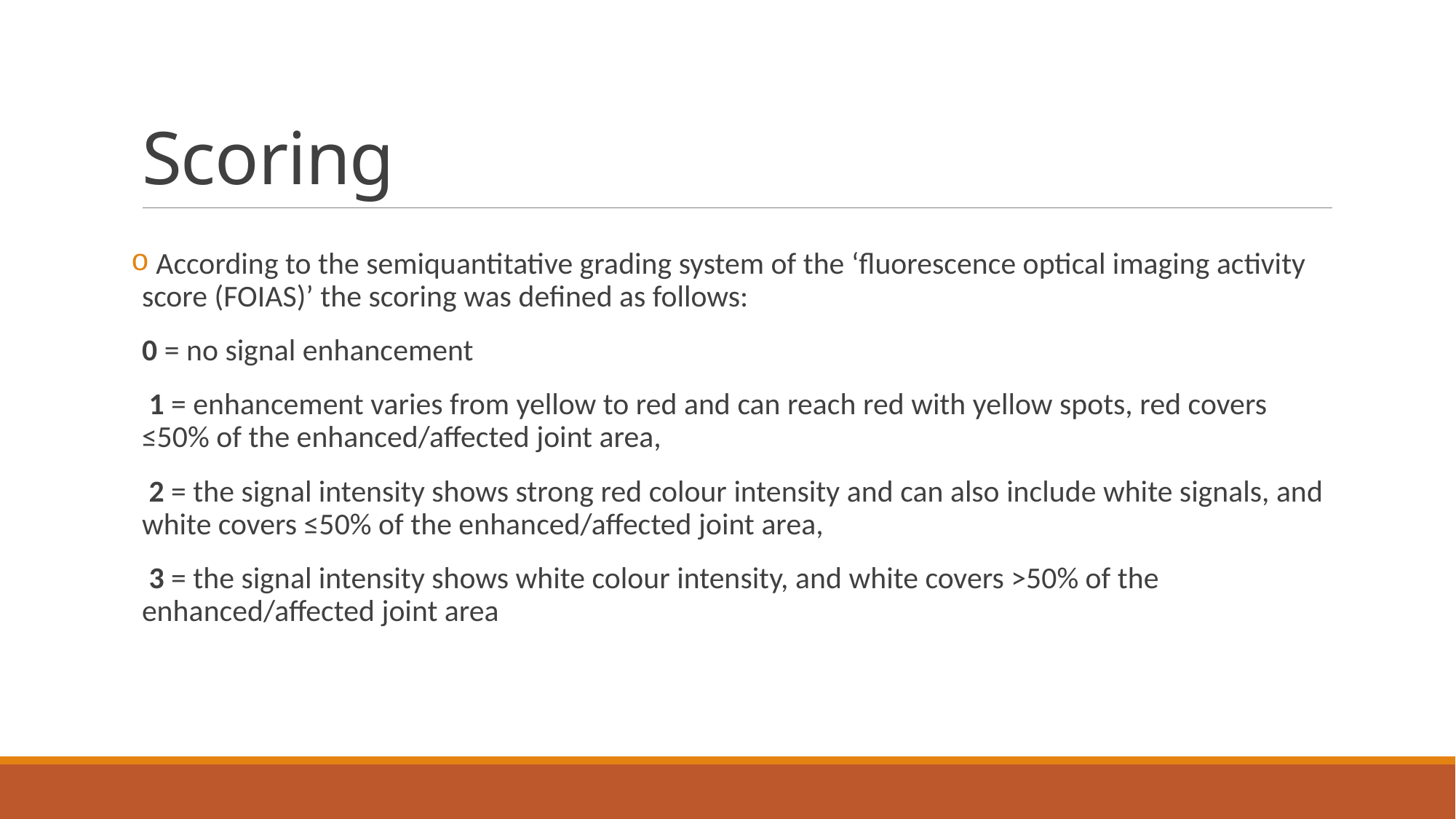

# Scoring
 According to the semiquantitative grading system of the ‘fluorescence optical imaging activity score (FOIAS)’ the scoring was defined as follows:
0 = no signal enhancement
 1 = enhancement varies from yellow to red and can reach red with yellow spots, red covers ≤50% of the enhanced/affected joint area,
 2 = the signal intensity shows strong red colour intensity and can also include white signals, and white covers ≤50% of the enhanced/affected joint area,
 3 = the signal intensity shows white colour intensity, and white covers >50% of the enhanced/affected joint area

## Slide 4
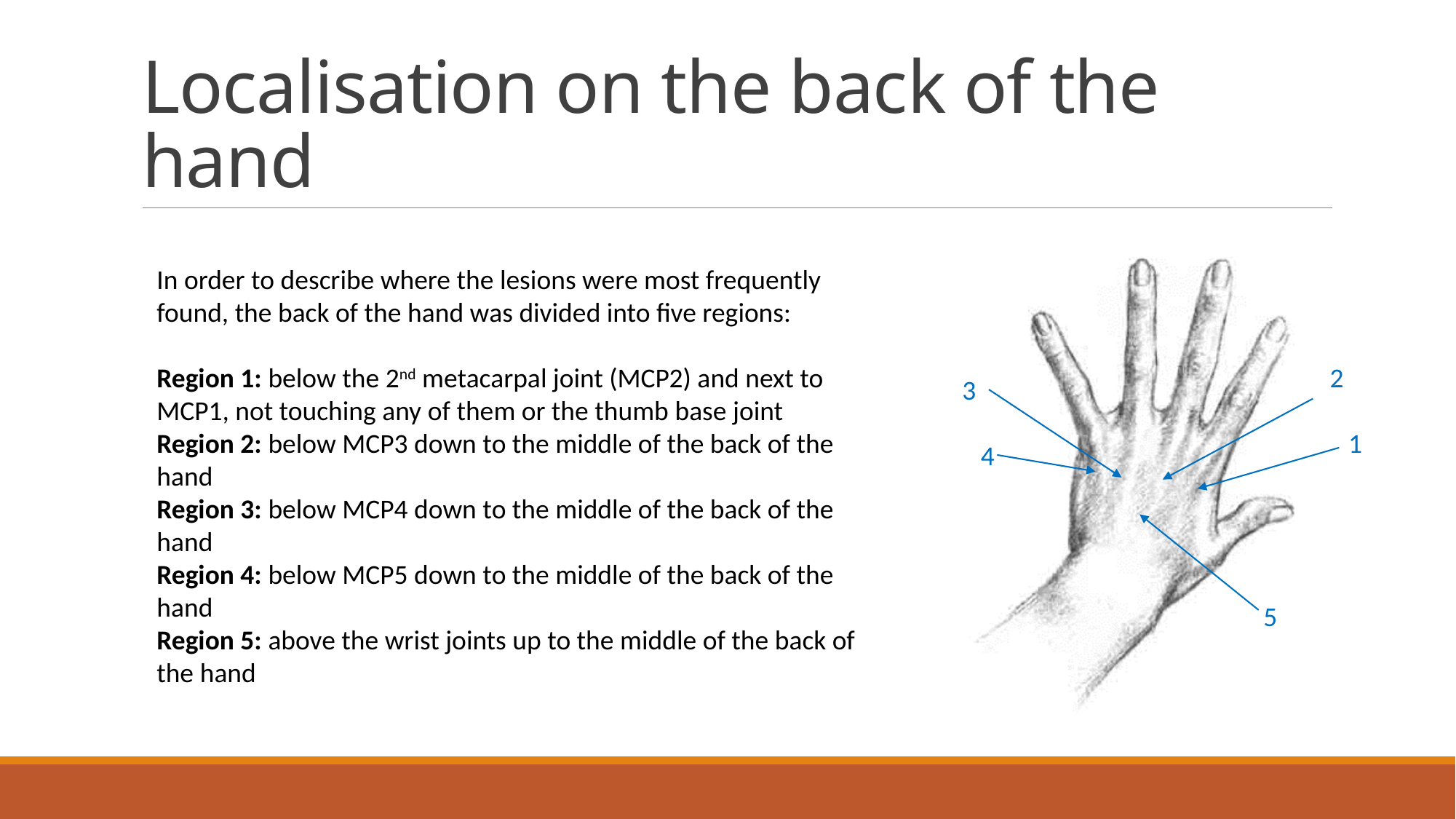

# Localisation on the back of the hand
 2
 1
In order to describe where the lesions were most frequently found, the back of the hand was divided into five regions:
Region 1: below the 2nd metacarpal joint (MCP2) and next to MCP1, not touching any of them or the thumb base joint
Region 2: below MCP3 down to the middle of the back of the hand
Region 3: below MCP4 down to the middle of the back of the hand
Region 4: below MCP5 down to the middle of the back of the hand
Region 5: above the wrist joints up to the middle of the back of the hand
 3
 4
5

## Slide 5
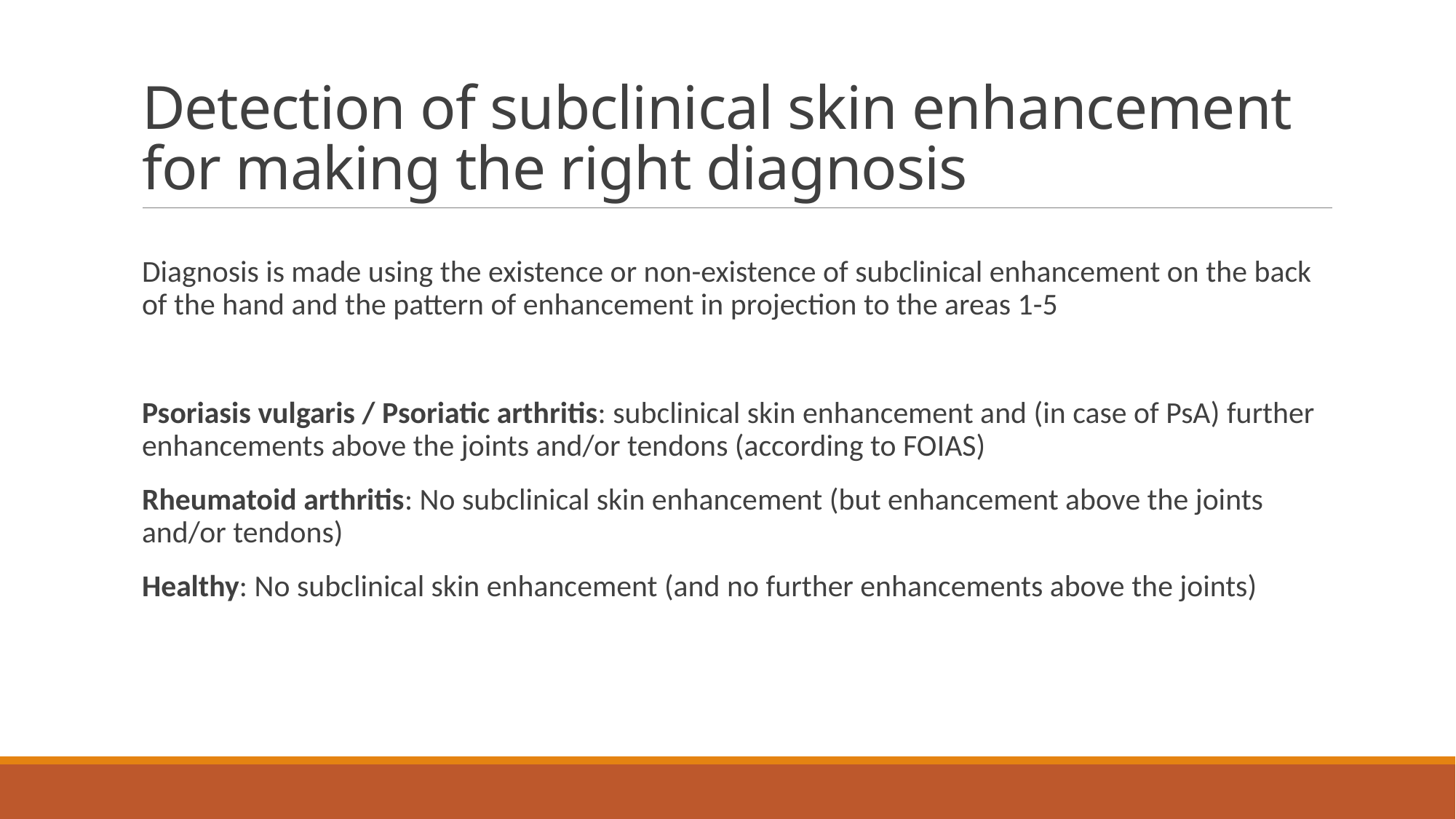

# Detection of subclinical skin enhancement for making the right diagnosis
Diagnosis is made using the existence or non-existence of subclinical enhancement on the back of the hand and the pattern of enhancement in projection to the areas 1-5
Psoriasis vulgaris / Psoriatic arthritis: subclinical skin enhancement and (in case of PsA) further enhancements above the joints and/or tendons (according to FOIAS)
Rheumatoid arthritis: No subclinical skin enhancement (but enhancement above the joints and/or tendons)
Healthy: No subclinical skin enhancement (and no further enhancements above the joints)

## Slide 6
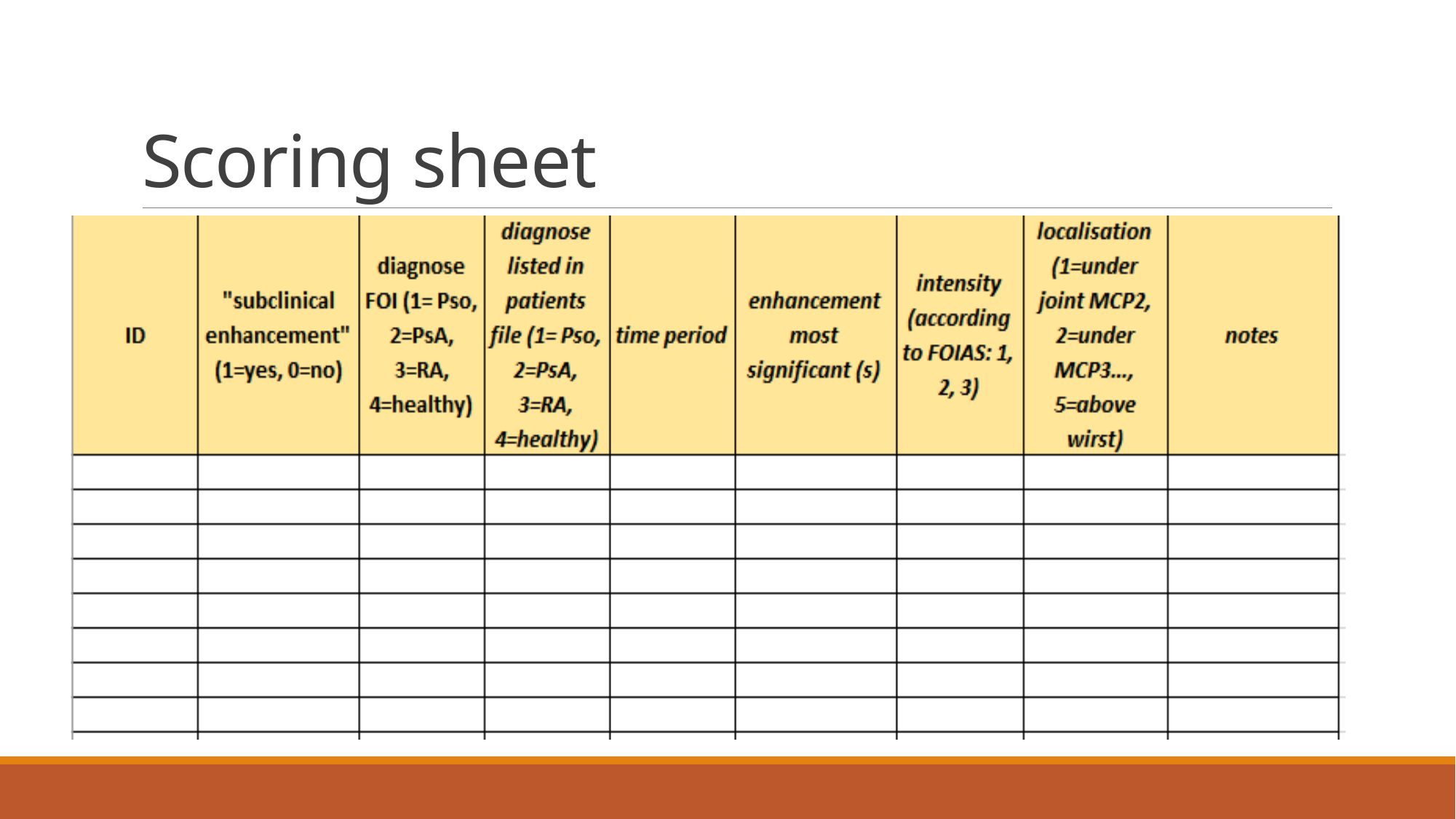

# Scoring sheet

## Slide 7
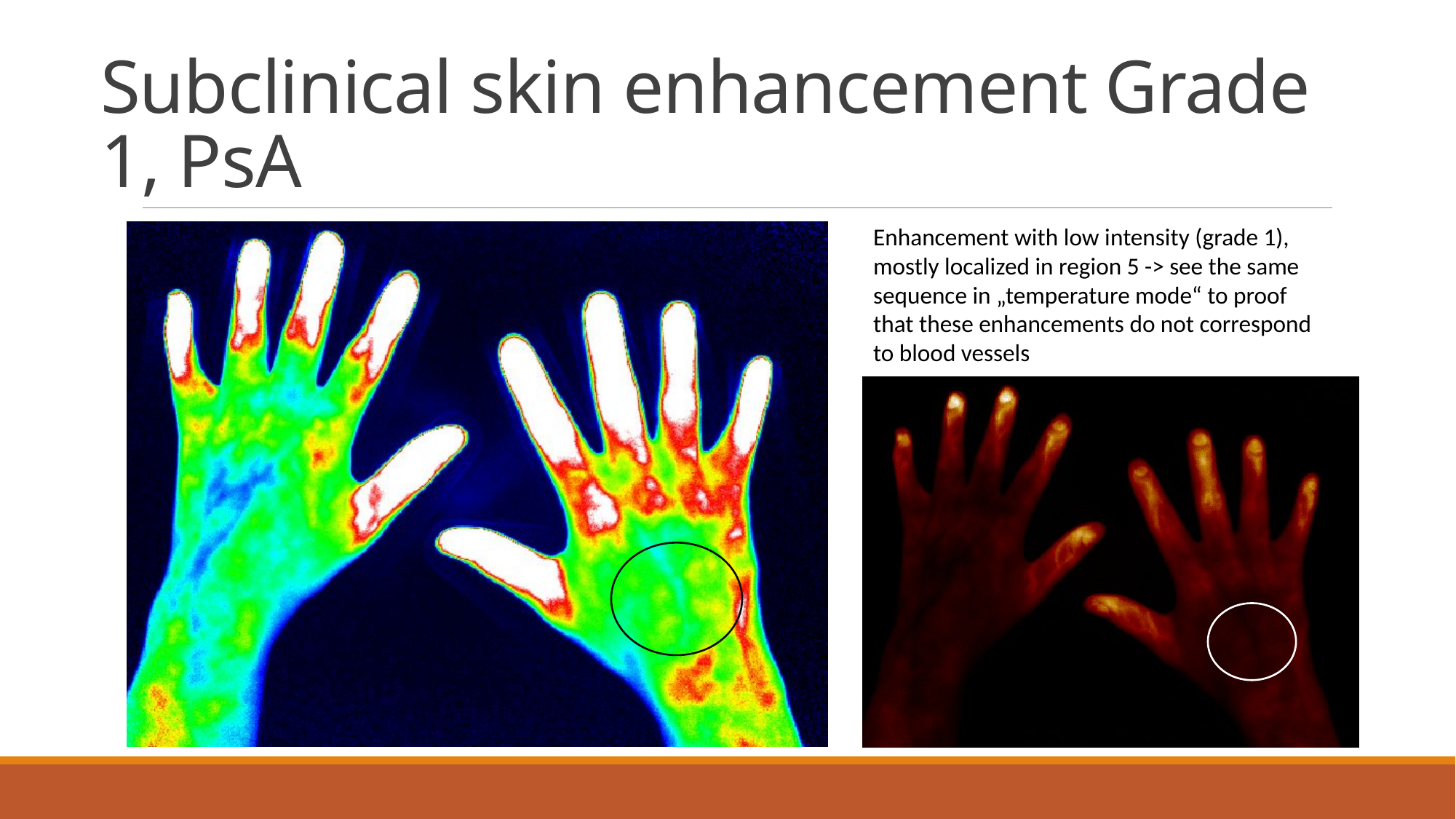

# Subclinical skin enhancement Grade 1, PsA
Enhancement with low intensity (grade 1), mostly localized in region 5 -> see the same sequence in „temperature mode“ to proof that these enhancements do not correspond to blood vessels

## Slide 8
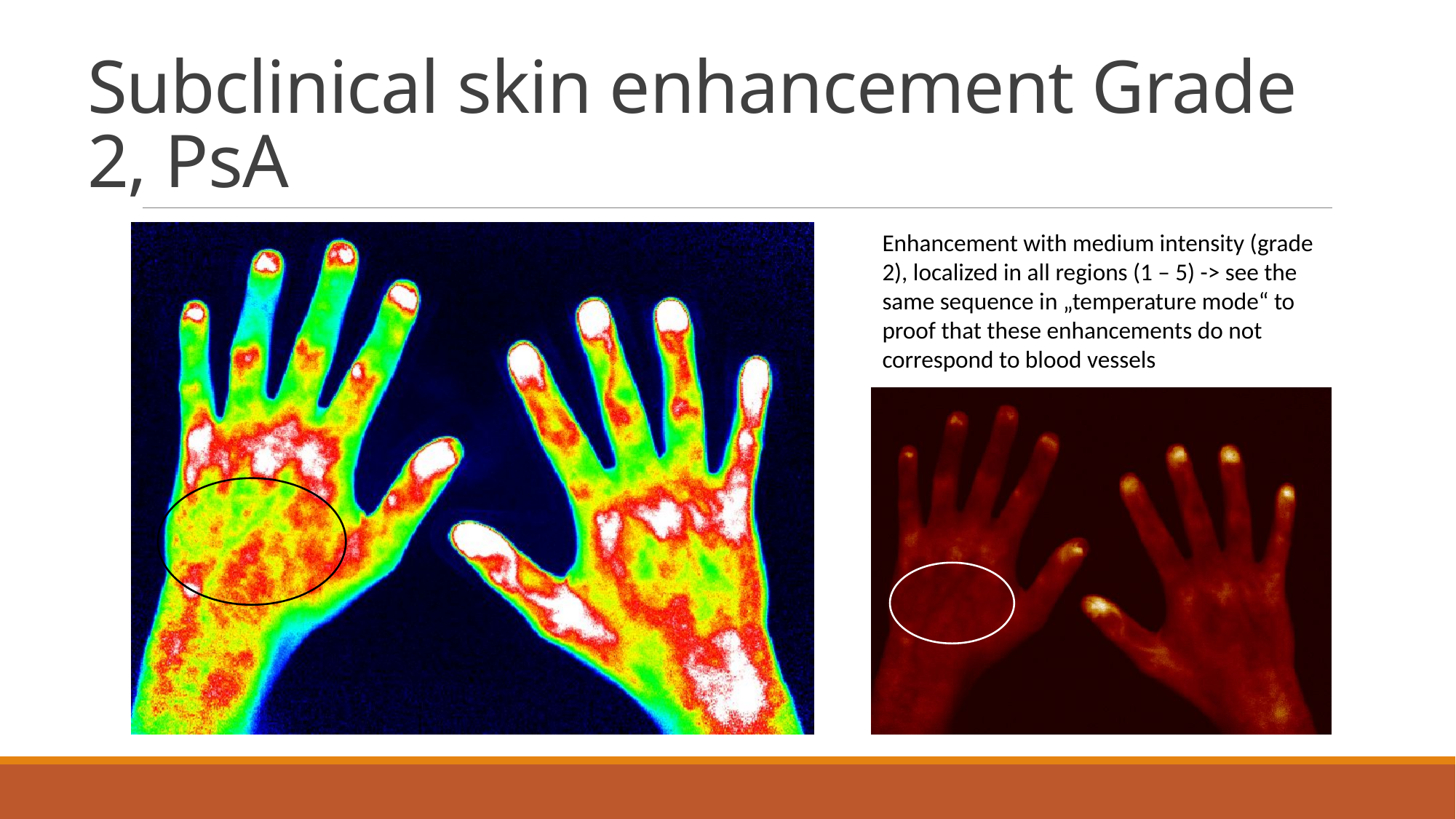

# Subclinical skin enhancement Grade 2, PsA
Enhancement with medium intensity (grade 2), localized in all regions (1 – 5) -> see the same sequence in „temperature mode“ to proof that these enhancements do not correspond to blood vessels

## Slide 9
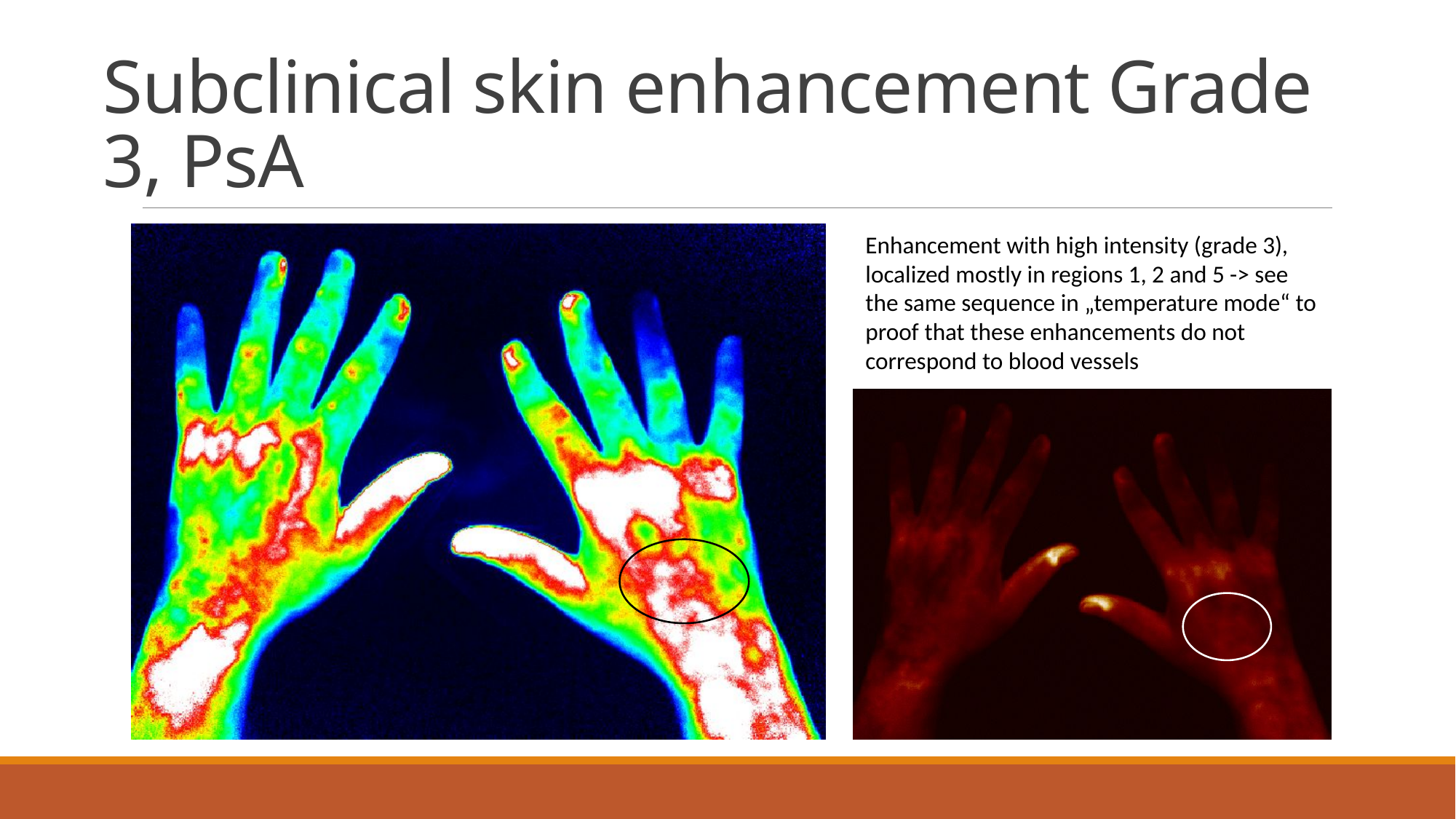

# Subclinical skin enhancement Grade 3, PsA
Enhancement with high intensity (grade 3), localized mostly in regions 1, 2 and 5 -> see the same sequence in „temperature mode“ to proof that these enhancements do not correspond to blood vessels

## Slide 10
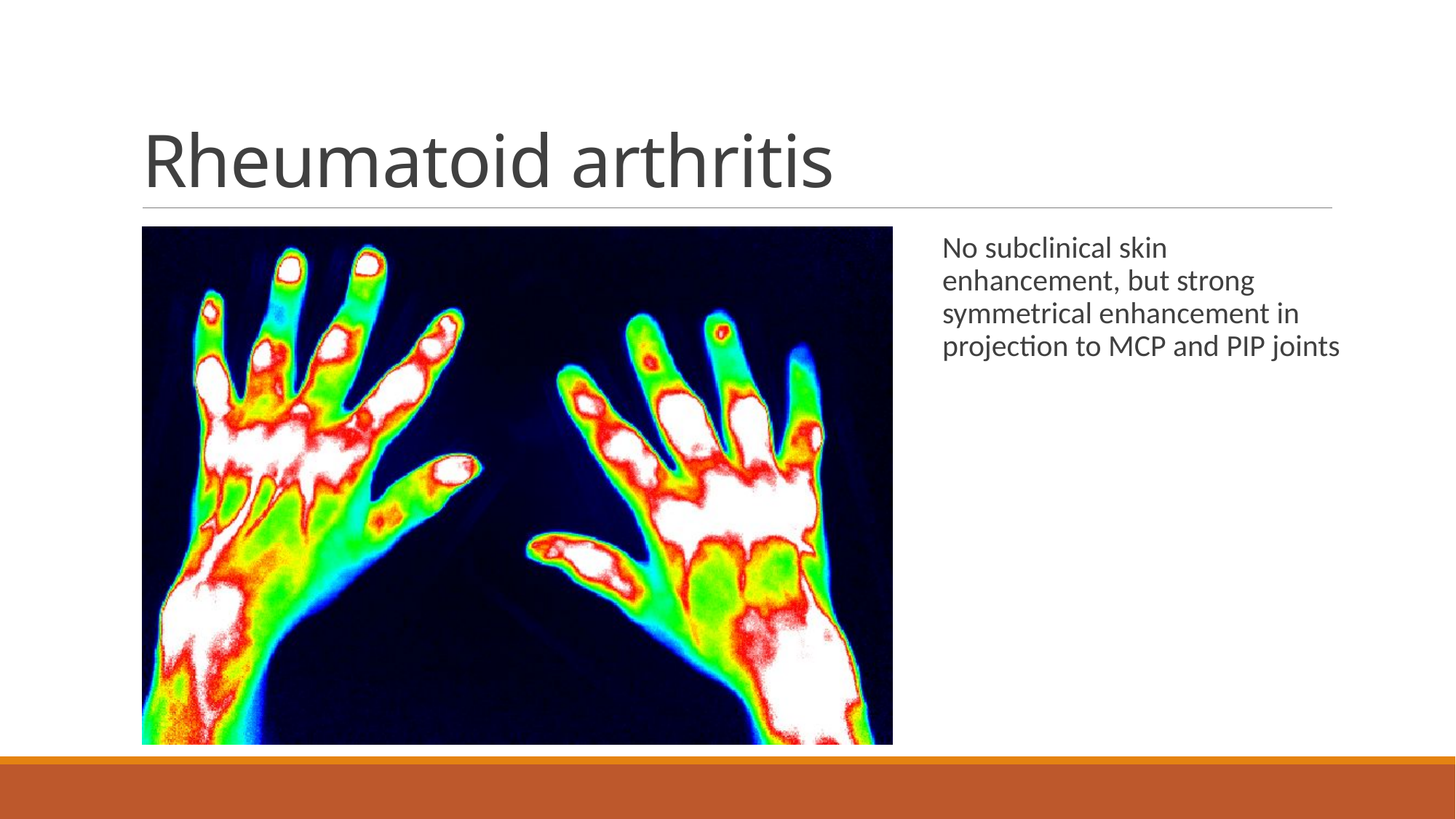

# Rheumatoid arthritis
No subclinical skin enhancement, but strong symmetrical enhancement in projection to MCP and PIP joints

## Slide 11
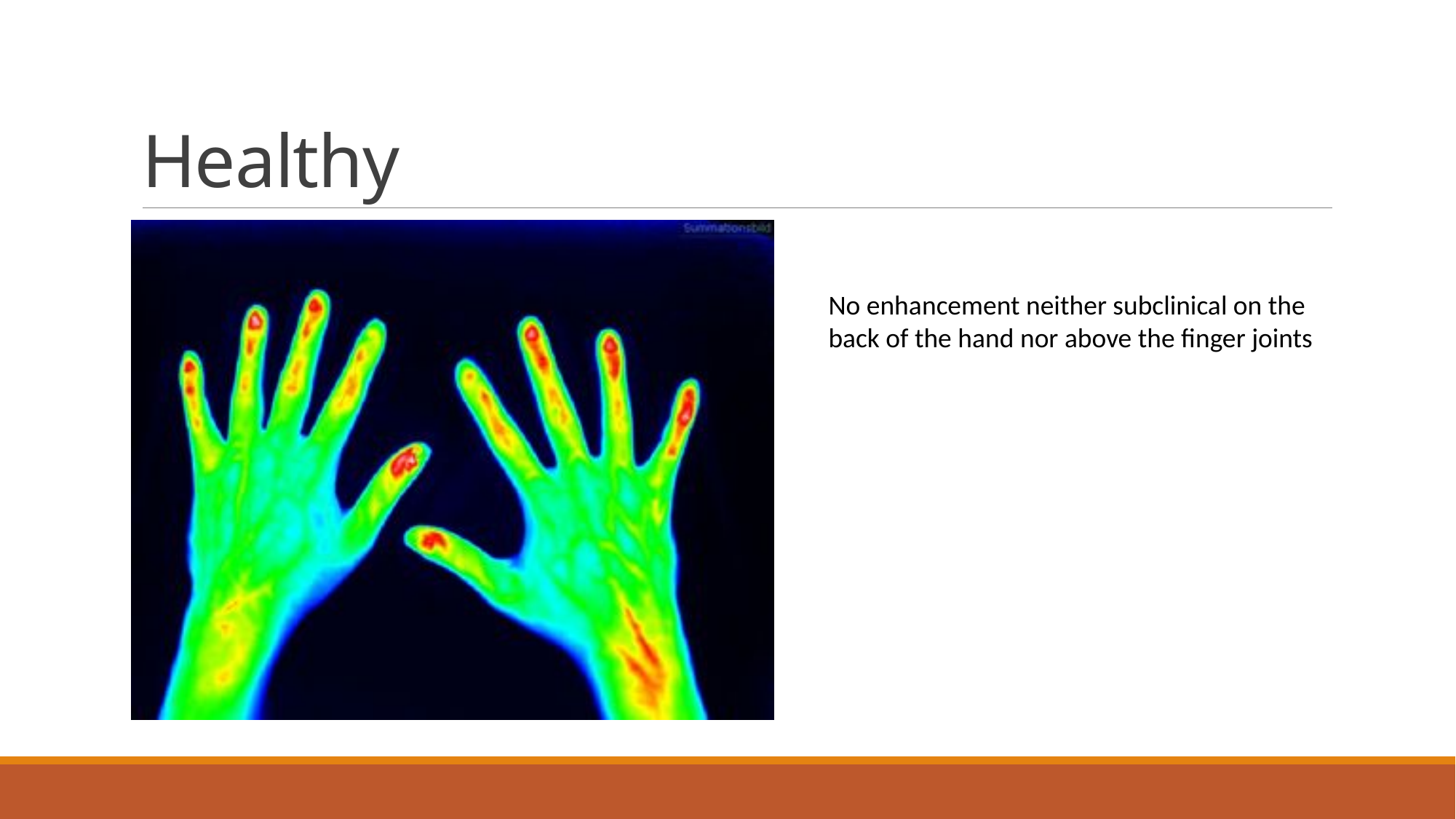

# Healthy
No enhancement neither subclinical on the back of the hand nor above the finger joints
